# Gaps in the screening process for women diagnosed with cervical cancer in four diverse US health care settings

**DOI:** 10.1002/cam4.5226

**Published:** 2022-09-15

**Authors:** Chun R. Chao, Jessica Chubak, Elisabeth F. Beaber, Aruna Kamineni, Connie Mao, Michael J. Silverberg, Jasmin A. Tiro, Celette Skinner, Michael Garcia, Douglas A. Corley, Rachel L. Winer, Tina Raine‐Bennett, Sarah Feldman, Cosette M. Wheeler

**Affiliations:** ^1^ Department of Research and Evaluation Kaiser Permanente Southern California Pasadena California USA; ^2^ Kaiser Permanente Washington Health Research Institute Seattle Washington USA; ^3^ Fred Hutchinson Cancer Research Center, Public Health Sciences Division Seattle Washington USA; ^4^ Department of Obstetrics and Gynecology, University of Washington Seattle Washington USA; ^5^ Division of Research Kaiser Permanente Northern California Oakland California USA; ^6^ Department of Population and Data Sciences University of Texas Southwestern Medical Center Dallas Texas USA; ^7^ Harold C. Simmons Comprehensive Cancer Center Dallas Texas USA; ^8^ Department of Epidemiology University of Washington School of Public Health Seattle Washington USA; ^9^ Division of Gynecologic Oncology, Brigham and Women's Hospital, Harvard Medical School Boston Massachusetts USA; ^10^ Center for HPV Prevention University of New Mexico Comprehensive Cancer Center Albuquerque New Mexico USA; ^11^ Medicines360 San Francisco California USA

**Keywords:** cancer screening, cervical cancer, cervical cancer prevention, cervical cancer screening, health service research

## Abstract

**Background:**

Potential care gaps in the cervical cancer screening process among women diagnosed with cervical cancer in an era with increased human papillomavirus (HPV) testing have not been extensively evaluated.

**Methods:**

Women diagnosed with cervical cancer between ages 21 and 65 at four study sites between 2010 and 2014 were included. Screening histories were ascertained from 0.5 to 4 years prior to cervical cancer diagnosis. We identified potential care gaps in the screening history for each woman and classified them into one of three mutually exclusive types: lack of a screening test, screening test failure, and diagnostic/treatment care gap. Distributions of care gaps were tabulated by stage, histology, and study site. Multivariable nominal logistic regression was used to examine the associations between demographic and cancer characteristics and type of care gap.

**Results:**

Of 499 women evaluated, 46% lacked a screening test in the time window examined, 31% experienced a screening test failure, and 22% experienced a diagnostic/treatment care gap. More than half of the women with advanced cancer and squamous cell carcinoma lacked a screening test compared to 31% and 24% of women with localized cancer and adenocarcinoma, respectively. Women aged 21–29 at diagnosis were more likely to experience screening test failure and diagnostic/treatment care gap, while those aged 50–65 were more likely to lack a screening test, compared to women aged 30–39.

**Conclusions:**

Our findings demonstrate a continuing need to develop interventions targeting unscreened and under‐screened women and improve detection and diagnosis of adenocarcinoma in women undergoing cervical cancer screening and diagnostic follow‐up.

## Graphical abstract

We examined potential care gaps in the cervical cancer screening process among 499 women diagnosed with cervical cancer in the era with increased human papillomavirus testing (2010‐2014). Of these women, 46% lacked a screening test in the time window examined, 31% experienced a screening test failure, and 22% experienced a diagnostic/treatment care gap. Our findings demonstrate a continuing need to develop interventions targeting unscreened and under‐screened women and improve detection and diagnosis in women undergoing screening and diagnostic follow‐up.

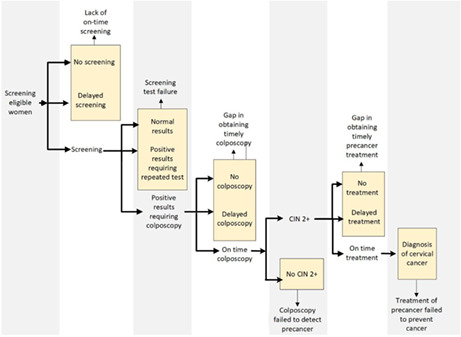

## 1 INTRODUCTION

Screening can prevent both incidence of and mortality from cervical cancer.^1^ The complete cancer screening process involves risk assessment, detection, diagnosis, and treatment.^2^ Care gaps in any of these steps can reduce the effectiveness of a cancer screening program. In the United States, approximately 12,000 women are diagnosed with cervical cancer annually.^3^ Prior to the introduction of human papillomavirus (HPV) testing, these cervical cancer diagnoses were found to be largely attributable to failure to screen followed by failure of the screening test (i.e., cytology test) to detect abnormal cells.^4^

US cervical cancer screening guidelines have moved from annual screening to longer screening intervals. More modality options are available in addition to cytology testing alone, including reflex HPV testing, concurrent cytology and HPV co‐testing,^5^ and, more recently, primary HPV testing.^6^ These guideline changes may have shifted the type of care gaps that contribute to cervical cancer development. Therefore, it is critical to provide data on care gaps in the cervical cancer screening process during the time period when HPV testing was increasingly used.

We examined screening, diagnostic follow‐up, and treatment history among women diagnosed with cervical cancer in diverse US health care settings between 2010 and 2014. Our objectives were to: (1) identify the most recent care gaps preceding diagnosis in the cervical cancer screening process; and (2) describe patient and cervical cancer characteristics associated with different types of care gaps across these diverse healthcare delivery settings.

## 2 METHODS

### 2.1 Study settings

This study was conducted as part of the National Cancer Institute‐funded Population‐based Research Optimizing Screening through Personalized Regimens (PROSPR I) consortium. The 10 PROSPR I study sites reflect the diversity of US delivery system organizations.^2^, ^7^ This study used data from the following four study sites contributing cervical cancer screening process data^8^: Kaiser Permanente Southern California (KPSC), Kaiser Permanente Northern California (KPNC), Parkland‐University of Texas Southwestern (Parkland‐UTSW), and the University of New Mexico (UNM). The health care settings and study populations for these PROSPR I sites have been described in detail previously.^8^ The PROSPR I Statistical Coordinating Center located at the Fred Hutchinson Cancer Research Center was responsible for data harmonization across the study sites, quality assurance, and statistical analyses. All study activities were approved by the Institutional Review Boards of the participating sites and the Statistical Coordinating Center and included waivers of informed consent.

For women ages 30 years and older, cytology with HPV co‐testing was adopted as the preferred screening strategy by KPNC and KPSC in 2003 and 2004, respectively. Co‐testing was introduced as an option that could be selected by providers at Parkland‐UTSW and in New Mexico in 2004. During the study period, cytology with reflex HPV testing was the preferred screening strategy at Parkland‐UTSW. The co‐testing proportion for screened women age 21–29 years ranged from a low of 4% in Parkland‐UTSW to a high of 18% in KPNC; for women age 30–65 years, it was >98% in KPSC/KPNC, 34% in the NMHPVPR, and 18% in Parkland‐UTSW. The HPV reflex testing proportion (among those screened with cytology) ranged from 4.5% in KPNC to 7.1% in the NMHPVPR for women age 21–29 years, and from 0% in KPSC and KPNC to 3.8% in Parkland‐UTSW for women age 30–65 years. The prevalence of HPV co‐testing and HPV reflex testing for each site was estimated using the method described by Cuzick *et al.*.^9^ HPV tests with genotyping information were used for screening for some women in New Mexico, but no other study site performed HPV genotyping during the study period.

### 2.2 Study population

The PROSPR I cervical study population for this analysis included women ages 18–89 between 2010 and 2013 at KPSC, KPNC, and Parkland‐UTSW; and between 2012 and 2014 in the NMHPVPR. At all sites, the study period preceded use of primary HPV testing. Cervical cancers diagnosed in the study populations were identified through linkage to local, state, or Surveillance, Epidemiology, and End Results (SEER) cancer registries. We restricted analyses to women who were 21–65 years old when diagnosed with invasive cervical cancer (i.e., within the recommended screening age range for average‐risk individuals). To evaluate screening history prior to cancer diagnosis, we further restricted to those with ≥4 years of health plan membership prior to cancer diagnosis at KPSC and KPNC and ≥4 years of engagement (i.e., active medical record) with Parkland‐UTSW.^10^ All women with a cervical cancer diagnosis who met the age criterion were included from the NMHPVPR.

### 2.3 Data collection

Information on age, race/ethnicity, cervical cancer diagnosis date, cervical cancer stage at diagnosis (SEER Summary Stage^11^), and cervical cancer histology was obtained from local, state, or SEER cancer registries. Information on cervical cancer screening tests (cytology and HPV), diagnostic and treatment procedures, and their results in the 4 years preceding the cervical cancer diagnosis were obtained from clinical and administrative data sources at KPSC, KPNC, and Parkland‐UTSW and from registry information systems at NMHPVPR. Cytology results were classified using the Bethesda system.^12^ All study sites used programmatic text string searches or natural language processing to extract diagnoses from pathology reports from biopsies, excisions, and relevant surgeries. If more than one histopathologic diagnosis was associated with a procedure, the most severe diagnosis was recorded for the procedure. Data sources and collection methods have been previously described in detail.^8^

### 2.4 Identification of potential care gaps in the screening history

**FIGURE 1:**
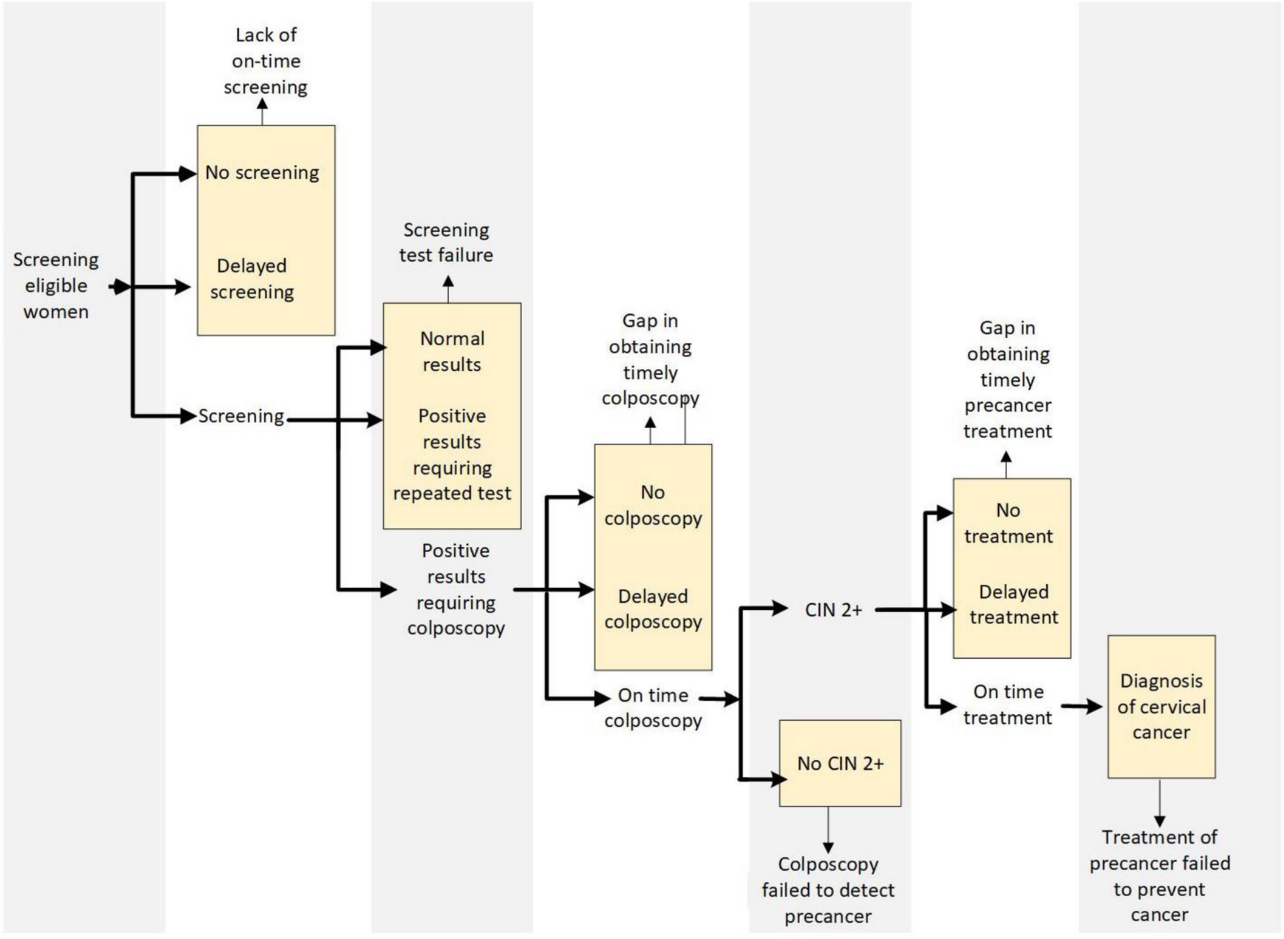
Gaps in the cervical cancer screening process in the presumed chronology of events. Care gaps were hierarchically assigned moving from right to left such that the most recent gap took precedence.

**FIGURE 2:**
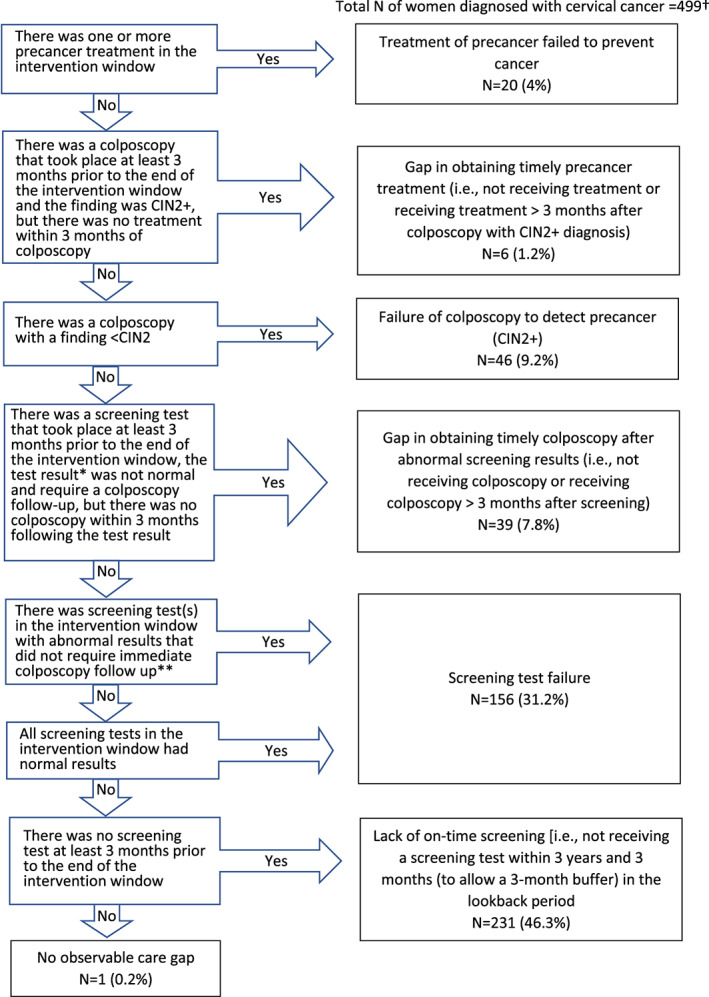
Algorithm for assigning failure type in the cervical cancer screening process. * Abnormal screening results defined as atypical squamous cell of undetermined significance (ASC‐US)/HPV+ or more severe result. The 2012 national cervical cancer screening guidelines were used to determine this for those who had Pap test alone, co‐testing or HPV reflex. Determination depends on age as per clinical guidelines. **Abnormal results that should receive follow‐up testing on alternate time intervals and not immediate colposcopy [i.e., NILM / high‐risk (hr) HPV positive or ASC‐US/ hrHPV negative] as per management guidelines for cervical abnormalities.13,14 The latter two abnormal results were considered screening test failures because findings may not have warranted an immediate colposcopy referral. Although some of these results may have been the second or third abnormal findings under the co‐testing or HPV reflex algorithm, they were treated equally as the first co‐testing/HPV reflex result for the purpose of this analysis given uncertainty in follow‐up regimens (i.e., they do not always trigger immediate colposcopy referral depending on if it is the first or second/repeated test). Further, the design of the lookback period in this study precluded determining if a result was from a repeat test. †Six women had missing results for Pap test and were excluded as it was not possible to correctly assign their care gap.

We examined potential care gaps in cervical cancer screening history in a “lookback period” from 6 months to 4 years prior to cervical cancer diagnosis (i.e., 3.5 years in length). Based on screening guidelines at the time,^13^, ^14^ women included in this study should have been screened for cervical cancer approximately every 1–3 years (depending on age and the specific screening modality). Thus, we expected to observe at least 1 screening test for each woman in the lookback period. The 6‐month period immediately prior to the cancer diagnosis was excluded to allow for diagnostic work up. Based on the natural history of cervical cancer, we assumed that a screening test during this window should have detected an abnormality. Thus, we sought to identify the most recent care delivery gap in the lookback period hierarchically as: (#1) treatment of precancer failed to prevent cancer; (#2) gap in obtaining timely precancer treatment; (#3) failure of colposcopy to detect precancer defined as cervical intraepithelial neoplasia grade 2 or greater (CIN2+); (#4) gap in obtaining timely colposcopy after abnormal screening results; (#5) screening test failure including screening results that were completely normal/negative or abnormal results that should receive follow‐up testing on alternate time intervals and not immediate colposcopy as per management guidelines^15^, ^16^; (#6) lack of on‐time screening; and (#7) no observable care gap. Detailed methods for identifying and assigning care gaps are provided in Figures 1 and 2, and Appendix S1.

These care gaps were further grouped into three types that corresponded to different domains of the cancer screening process: (I) lack of a screening test, (II) screening test failure, and (III) diagnostic/treatment care gap. These types correspond to care gaps #6, #5, and #1–4 above, respectively.

### 2.5 Statistical analysis

We compared demographic and clinical characteristics for women with cervical cancer who were included vs. excluded from the study. Among those included, we examined the distributions of demographic and clinical characteristics. We then examined the distributions of the care gaps by stage at diagnosis [SEER summary stage localized (FIGO [International Federation of Gynecology and Obstetrics] stage I) versus advanced stages (regional (FIGO stage II‐III) and distant stages (FIGO stage IV))], histology, and study site.

We estimated the crude and adjusted associations between demographic and clinical characteristics and the most recent care gap type as the outcome of interest. Multivariable nominal logistic regression was performed, mutually adjusted for year of diagnosis, age at diagnosis, race/ethnicity, stage at diagnosis, histology, and study site. The models result in two odds ratios for each exposure with the outcomes of (1) “diagnostic/treatment care gap” and (2) “screening test failure”, with each compared to the “lack of a screening test” outcome. All analyses were conducted using the statistical package R, version 3.5.2.^17^

## 3 RESULTS

A total of 969 cervical cancers were diagnosed at the four study sites during the study period; 505 met the inclusion criteria. Across sites, the majority (59%) of cancers were diagnosed at a localized stage (FIGO stage I) and a majority were squamous cell carcinomas (SCC) (Table 1). Adenocarcinomas comprised 35% of the cancers. Comparison of characteristics for the 505 women included versus 464 women excluded from the study were similar across study sites, except for age (younger women were less likely to have ≥4 years of prior health plan membership/engagement compared with older women, data not shown).

**TABLE 1:** Characteristics of women diagnosed with cervical cancer between ages 21–65 years at PROSPR I cervical sites

		Site 1	Site 2	Site 3	Site 4	Total
Cases diagnosed		*N* = 152	*N* = 113	*N* = 78	*N* = 162	*N* = 505
Diagnosis year	2010–2012	117 (77.0%)	90 (79.6%)	48 (61.5%)	57 (35.2%)	312 (61.8%)
	2013–2014	35 (23.0%)	23 (20.4%)	30 (38.5%)	105 (64.8%)	193 (38.2%)
Age at diagnosis (years)	21–29	6 (3.9%)	<5^a^	<5^a^	20 (12.4%)	33 (6.5%)
	30–39	33 (21.7%)	26 (23.0%)	29 (37.2%)	37 (22.8%)	125 (24.8%)
	40–49	44 (28.9%)	38 (33.6%)	18 (23.1%)	44 (27.2%)	144 (28.5%)
	50–59	51 (33.6%)	26 (23.0%)	22 (28.2%)	45 (27.8%)	144 (28.5%)
	60–65	18 (11.8%)	20 (17.7%)	5 (6.4%)	16 (9.9%)	59 (11.7%)
Race/ethnicity	Non‐Hispanic White	55 (36.2%)	70 (62.0%)	16 (20.5%)	73 (45.1%)	214 (42.4%)
	Non‐Hispanic Black	14 (9.2%)	8 (7.1%)	23 (29.5%)	<5^a^	47 (9.3%)
	Hispanic	56 (36.8%)	19 (16.8%)	36 (46.2%)	69 (42.6%)	180 (35.6%)
	Asians/Pacific Islander	25 (16.4%)	15 (13.3%)	<5^a^	<5^a^	45 (8.9%)
	Other/Unknown	<5^a^	<5^a^	<5^a^	15 (9.3%)	19 (3.8%)
SEER summary stage at diagnosis^b^	Localized	94 (61.8%)	78 (69.0%)	40 (51.3%)	88 (54.3%)	300 (59.4%)
	Regional	47 (30.9%)	21 (18.6%)	23 (29.5%)	49 (30.3%)	140 (27.7%)
	Distant	11 (7.2%)	13 (11.5%)	11 (14.1%)	19 (11.7%)	54 (10.6%)
	Unknown	0 (0%)	<5^a^	<5^a^	6 (3.7%)	11 (2.2%)
Histology	Squamous cell carcinoma	87 (57.2%)	47 (41.6%)	59 (75.6%)	110 (67.9%)	303 (60.0%)
	Adenocarcinoma	61 (40.1%)	57 (50.4%)	15 (19.3%)	42 (25.9%)	175 (34.7%)
	Other	4 (2.6%)	9 (8.0%)	<5^a^	10 (6.2%)	27 (5.3%)
Health insurance type	Commercial/private	143 (94.1%)	95 (84.2%)	<5^a^	NA	240 (47.5%)
	Medicaid	<5^a^	<5^a^	28 (35.9%)	NA	33 (6.5%)
	Medicare	6 (3.9%)	16 (14.2%)	<5^a^	NA	25 (5.0%)
	Uninsured	0 (0%)	0 (0%)	22 (28.2%)	NA	22 (4.4%)
	Other/Unknown	0 (0%)	0 (0%)	23 (29.5%)	162 (100%)	185 (36.6%)

Abbreviations: NA, Information not available; PROSPR I, Population‐based Research Optimizing Screening through Personalized Regimens‐1; SEER, Surveillance, Epidemiology, and End Results.

a Actual number not shown when cell size <5 for site‐specific data.

b SEER Summary Stage Localized = FIGO stage I, Regional = FIGO stage II and III, Distant = FIGO stage IV.

### 3.1 Distribution of potential care gaps overall and by stage at diagnosis

A total of 499 women were assigned care gaps (6 women had missing cytology results thus their care gap could not be assigned). Overall, 46% did not have a screening test in the lookback window, 31% had a screening test failure, while 22% experienced a diagnostic/treatment care gap (Table 2). Women diagnosed with advanced stage cancer predominantly lacked a screening test in the lookback period (70%), while 20% had a screening test failure and 10% had a diagnostic/treatment care gap. Of note, 26% of those diagnosed at distant stage experienced a “screening test failure” (i.e., a test with results that would not trigger immediate colposcopy referral). Among women with localized cancer, 31% did not have a screening test in the lookback period, 38% had a screening test failure, while 30% experienced a diagnostic/treatment care gap.

**TABLE 2:** Distribution of potential care gaps and care gap types in the screening history among women diagnosed with cervical cancer by stage at diagnosis and histology

	By stage at diagnosis					By histology			
	Advanced stages								
Care gap type specific care gap	Total *N* = 191	Regional *N* = 137	Distant *N* = 54	Localized *N* = 297	Unknown *N* = 11	Squamous cell carcinoma *N* = 299	Adenocarcinoma *N* = 173	Other *N* = 27	Total *N* = 499^a^
Diagnostic/treatment care gap	**19 (9.9%)**	**16 (11.7%)**	**3 (5.6%)**	**90 (30.3%)**	**2 (18.2%)**	**56 (18.7%)**	**52 (30.1%)**	**3 (11.1%)**	**111 (22.2%)**
Treatment of precancer failed to prevent cancer	4 (2.1%)	4 (2.9%)	0 (0%)	16 (5.4%)	0 (0%)	11 (3.7%)	9 (5.2%)	0 (0%)	20 (4.0%)
Gap in obtaining precancer treatment	1 (0.5%)	1 (0.7%)	0 (0%)	5 (1.7%)	0 (0%)	5 (1.7%)	1 (0.6%)	0 (0%)	6 (1.2%)
Colposcopy failed to detect precancer	5 (2.6%)	4 (2.9%)	1 (1.9%)	40 (13.5%)	1 (9.1%)	16 (5.4%)	28 (16.2%)	2 (7.4%)	46 (9.2%)
Gap in follow‐up with colposcopy	9 (4.7%)	7 (5.1%)	2 (3.7%)	29 (9.8%)	1 (9.1%)	24 (8.0%)	14 (8.1%)	1 (3.7%)	39 (7.8%)
Screening test failure	**39 (20.4%)**	**25 (18.2%)**	**14 (25.9%)**	**114 (38.4%)**	**3 (27.3%)**	**72 (24.1%)**	**79 (45.6%)**	**5 (18.5%)**	**156 (31.2%)**
Lack a screening test	**133 (69.6%)**	**96 (70.1%)**	**37 (68.5%)**	**92 (31.0%)**	**6 (54.5%)**	**170 (56.9%)**	**42 (24.3%)**	**19 (70.4%)**	**231 (46.3%)**
No clear care gap	**0 (0%)**	**0 (0%)**	**0 (0%)**	**1 (0.3%)**	**0 (0%)**	**1 (0.3%)**	**0 (0%)**	**0 (0%)**	**1 (0.2%)**

*Note*: The percentage for the care gap types represents the sum of the percentage of the care gaps within that type. Percentages of the care gap types (in bold) add to 100%.

a Six women had missing results for Pap test and were excluded from Table 2 as it was not possible to correctly assign their care gap.

### 3.2 Distribution of potential care gaps by histology and study site

When we examined the care gap distribution by histology, only a quarter of the women with adenocarcinoma lacked a screening test compared with 57% of the women with SCC (Table 2). In contrast, a larger proportion of women with adenocarcinoma experienced a screening test failure (46% vs. 24% of women with SCC) and diagnostic/treatment care gap (30% vs. 19% of women with SCC).

Care gap distributions also varied significantly by study site (Table S1). Sites 1 and 2 had similar proportions of women in all three types of care gaps. However, at sites 3 and 4, lack of a screening test in the lookback period was the dominant type of care gap, while diagnostic/treatment care gaps were less common.

### 3.3 Distribution of screening modality and results among those with “screening test failure”

Of the 156 women who experienced a screening test failure, 59 had a cytology test only and 97 had a co‐test. Among the 97 women with a co‐test, the results were negative/unsatisfactory cytology and negative HPV test [*n* = 63 (65%); 2 with unsatisfactory cytology], negative/unsatisfactory cytology test and positive HPV test [*n* = 27 (28%); 1 with unsatisfactory cytology], and ASC‐US and negative HPV test [*n* = 6 (6%)]. One woman (6%) had ASC‐US and positive HPV which did not require immediate colposcopy due to young age.

### 3.4 Characteristics associated with care gap types among women diagnosed with cervical cancer

In multivariable analyses, women diagnosed at ages 21–29 were more likely to have experienced a “screening test failure” and “diagnostic/treatment care gap” rather than “lack of a screening test” compared to women diagnosed at ages 30–39: odds ratios (ORs) and 95% confidence intervals (95% CIs) for age 21–29 years versus 30–39 years = 3.58 (1.18–10.80) and 3.43 (1.04–11.30), respectively (Table 3). In contrast, opposite associations were observed for women diagnosed at ages 50–65. Non‐Hispanic Black and Asian/Pacific Islander women with cancer were more likely to have experienced a “screening test failure” and “diagnostic/treatment care gap” rather than “lack of a screening test” compared to non‐Hispanic White women with cancer. Women diagnosed with advanced stages were less likely to have experienced a screening test failure or diagnostic/treatment care gap than women with localized cancer. Women with adenocarcinoma were more likely to have experienced a screening test failure or diagnostic/treatment care gap than women with SCC (Table 3).

**TABLE 3:** Adjusted odds ratios from multivariable nominal logistic regression for care gap type among women diagnosed with cervical cancer

	Screening test failure (*N* = 156)^a^	Diagnostic/treatment care gap (*N* = 111)^a^
	Comparison group for outcomes: Lack of a screening test (*N* = 231)^a^	
	Odds Ratio (95% CI)^b^	
Diagnosis year		
2010–2012	Ref	Ref
2013–2014	1.42 (0.83–2.43)	1.66 (0.89–3.08)
Age at diagnosis (years)		
21–29	3.58 (1.18–10.80)	3.43 (1.04–11.30)
30–39	Ref	Ref
40–49	0.77 (0.40–1.49)	0.73 (0.36–1.51)
50–59	0.48 (0.24–0.93)	0.34 (0.15–0.73)
60–65	0.38 (0.15–0.93)	0.35 (0.13–0.96)
Race/ethnicity		
Non‐Hispanic White	Ref	Ref
Non‐Hispanic Black	2.64 (1.07–6.47)	2.85 (1.00–8.13)
Hispanic	1.65 (0.93–2.93)	1.55 (0.82–2.96)
Asian/Pacific Islander	2.67 (1.07–6.67)	2.75 (1.02–7.38)
Other/Unknown	0.62 (0.17–2.24)	0.34 (0.06–1.90)
SEER summary stage at diagnosis		
Localized	Ref	Ref
Regional	0.19 (0.11–0.35)	0.14 (0.07–0.29)
Distant	0.50 (0.23–1.11)	0.15 (0.04–0.55)
Unknown	1.31 (0.25–6.79)	1.62 (0.24–11.10)
Histology		
Squamous cell carcinoma	Ref	Ref
Adenocarcinoma	3.91 (2.31–6.61)	3.12 (1.73–5.62)
Other	0.42 (0.13–1.34)	0.38 (0.09–1.66)
Study site		
Site 1	1.30 (0.65–2.58)	1.75 (0.83–3.67)
Site 2	Ref	Ref
Site 3	0.25 (0.11–0.59)	0.10 (0.03–0.32)
Site 4	0.35 (0.17–0.73)	0.30 (0.13–0.69)

Abbreviations: CI, confidence interval; Ref, reference group; SEER, Surveillance, Epidemiology, and End Results.

a One woman without a clear care gap and 6 women with missing results for Pap test (thus it was not possible to correctly assign their care gap) were excluded, leaving 498 women in this analysis.

b Model adjusted for all variables shown in this table. We did not include insurance type in this analysis due to inability to obtain stable estimates for insurance type. Additionally, adjusting for insurance type did not lead to material changes of the results presented in this table.

## 4 DISCUSSION

In interpreting our results, it is important to consider several features of the study design. First, every woman had cervical cancer and was assigned a potential care gap. Thus, the mutually exclusive care gap categories sum to 100%. Our results should be interpreted as such and not as underlying rates of care gaps among all women undergoing screening. Second, the goal of the study was not to make a causal inference about which gaps, if any, lead to cancer. This was not a study of screening effectiveness; such a study would require control groups. Third, because the lookback period was only up to 3.5 years in length, we could only examine the most recent care gap. Additionally, early detection of cervical cancer can be considered a success; however, because it is preventable by screening, we included all stages of invasive cancer in this analysis. With these considerations in mind, our study still provides important information on understanding gaps in cervical cancer screening delivery.

In our study, 46% of women diagnosed with cervical cancer lacked a recent screening test in the lookback period, 31% experienced a screening test failure, and 22% experienced a diagnostic/treatment failure care gap. Thus, gaps in obtaining recent screening remained a common phenomenon among women diagnosed with cervical cancer, especially among those diagnosed at advanced stages, despite decades of widespread cervical cancer screening in the United States. About 70% of women diagnosed with localized cervical cancer in this study experienced either a screening test failure or diagnostic/treatment care gap. These data suggest that interventions other than those related to screening uptake are worth exploring.

Most prior studies have reported screening process failures in women diagnosed with cervical cancer before co‐testing with cytology and HPV tests was widely adopted. Spence and colleagues systematically reviewed studies from Europe, the United States, and Australia between 1950 and 2007^18^ and reported that, on average, 54% of women with cervical cancer did not have an adequate screening history. Among those screened, an estimated 29% experienced at least one false‐negative cytology result, and among those with an abnormal screening result, 12% had inadequate follow‐up. Significant heterogeneity in results was observed in prior studies across countries, study settings, and calendar years.

In our study, a considerable proportion of women experienced a screening test failure, including those from study sites where co‐testing was performed in almost 100% of the women in the recommended age range. Notably, a large proportion (65%) of women screened by co‐testing who experienced a screening test failure had both negative cytology and HPV test (which could be due to inadequate sampling not recognized initially), while most of the remaining women had a negative cytology test and a positive HPV test. A prior study by Castle and colleagues examined screening histories of women diagnosed with cervical cancer at KPNC after the implementation of co‐testing.^19^ They also observed that a considerable proportion of these women experienced diagnostic delays due to the need for repeat testing of specific co‐testing results (e.g., negative Pap test and a positive HPV test). For some of these women, triaging based on HPV genotypes (i.e., sending women with HPV 16/18 directly to colposcopy) may mitigate the delay due to the repeated testing algorithm recommended with co‐testing.^20^, ^21^ In addition, 45% of the women with cervical adenocarcinoma experienced a screening test failure in our study. This is consistent with previous research.^4^, ^10^, ^18^ A cytology test is not as sensitive for detecting adenocarcinoma in situ (AIS) or adenocarcinoma.^22^, ^23^ It should also be noted that in our study 26% of women with distant stage cancer experienced a screening test failure. It is possible that some of these are missed by the evaluators or obscured samples due to the presence of blood and necrosis. In a recent study, variations in sampling and cytology interpretation each accounted for about half of the false‐negative Pap tests.^24^

Taken together, these data suggest the need to further improve screening coverage and detection of cervical precancers. A recent study reported that lack of knowledge was the biggest barrier for getting timely screening among under‐screened women.^25^ To this end, educational media campaigns and physician communications/outreach have been shown effective in improving screening coverage.^26^ With updated guidelines recommending primary HPV screening, home‐based self‐collection for HPV testing may be a promising approach to screen women who are unable or prefer not to have a clinician collected sample.^27^, ^28^, ^29^ Beyond screening uptake, new techniques such as dual stain and methylation may help risk stratify patients with abnormal screening results to colposcopy and biopsy.^30^, ^31^, ^32^, ^33^ New studies have supported enhanced biopsy practice (e.g., random four‐quadrant biopsies) to improve detection,^34^, ^35^ especially for adenocarcinoma.^36^ The development of prediction models may help optimize the use of endocervical curettage in clinical practice. Accelerating the uptake of latest practice guidelines and validated new tools/technology by community‐based practices will be critical to further reduce the incidence of cervical cancer.

We also observed varying distributions of care gaps among women diagnosed with cervical cancer across the study sites. The differences observed may reflect heterogeneity across study sites in cervical cancer screening practices (i.e., differences in the screening modalities used, guidelines followed, presence of an organized screening program, level of in‐/outreach by the healthcare system), study eligibility criteria (i.e., enrollment vs. non enrollment‐based sites which may affect data completeness), and differing characteristics of the populations served across study sites.^8^

Women diagnosed at age 50–65 years were more likely to lack a screening test (rather than the other care gaps) in the lookback period compared to women diagnosed at age 30–39 years. Although we did not evaluate screening rates, this association is consistent with the literature observing that screening rates decline with age.^37^ A recent study based on women diagnosed with cervical cancer in New Mexico also reported similar findings for women age 45–64.^38^ Conversely, women ages 21–29 were more likely to have experienced care gaps in detection and diagnosis/treatment (rather than lack of screening). This observation may be partially explained by different screening approaches for this age group (i.e., cytology only or cytology with HPV reflex), and the generally less aggressive management strategies for detected abnormalities.^39^ Further, we observed that non‐Hispanic Black and Asian/Pacific Islander women diagnosed with cervical cancer more often experienced a screening test failure and/or a diagnostic/treatment care gap, and thus less often lacked a screening test. The finding for Asian/Pacific Islander women with cervical cancer is somewhat in contrast to the national reports of lower cytology screening rates among Asian women compared with non‐Asian women.^40^ As most Asian/Pacific Islander women in this study were KPNC and KPSC members, it is possible that Asian/Pacific Islander women may not be less likely to screen when they are insured and/or when there are centralized reminders. The findings for non‐Hispanic Black women in this study are generally consistent with several previous studies that reported that Black women were less likely to receive diagnostic procedures after an abnormal screening result compared to white women.^41^, ^42^ Specific barriers for diagnostic follow‐up for non‐Hispanic black and Asian/Pacific Islander women should be further identified to inform design of effective interventions.

There are several limitations that should be considered when interpreting the study results. In addition to those noted above, our relatively short (3.5‐year) lookback period precluded a more detailed evaluation of care gaps for certain follow‐up algorithms of co‐testing or HPV reflex. Monitoring screening patterns over longer periods is challenging due to people's movement across health care settings in the US^43^, ^44^ and such data were unavailable for our study population. Second, 40% of the women diagnosed with cervical cancer during the study period were excluded from the analysis due to lack of sufficient clinical history for review. Our findings may not represent the experiences of women who are engaged with healthcare systems for shorter periods of time and who are uninsured. Further, misclassification of the care gap in women in the NMHPVPR may be possible because we were unable to identify and exclude women who did not have a full 4‐year residential history in New Mexico prior to cancer diagnosis. Third, our definition of care gap within the lookback period may not be 100% accurate for highly aggressive, fast progressing cancers (e.g., a pre‐cancer lesion may not be present in the lookback period), although this is expected to be rare. Fourth, the sample size was small for certain comparisons (e.g., women diagnosed between ages 21–29), resulting in somewhat wide confidence intervals. Fifth, we were unable to distinguish tests that were done for surveillance versus routine screening. Finally, this study was conducted prior to the release of guidelines recommending primary HPV testing which has had a slow adoption in the US.^6^ Future studies should continue to monitor if distribution of care gaps shifts with more widespread implementation of this modality and more recent changes in management guidelines.

Despite these limitations, our study has several unique strengths. We are among the first to describe recent screening care gaps in cervical cancer cases diagnosed in diverse health care settings in an era with increased HPV testing. Additionally, the detailed algorithms used to understand gaps in each step of the screening process can be readily adopted by other healthcare settings with varying screening protocols.

## 5 CONCLUSION

The effectiveness of population‐based cervical cancer screening, including screening coverage, test performance, and timely follow‐up care continue to deserve attention to further prevent cervical cancer. Our results indicate that screening adherence and timely diagnostic evaluation may represent opportunities for quality improvement. Future quantitative and qualitative research is needed to better understand and address heterogeneity in the cervical cancer screening process across diverse US health care settings and potential disparities across age groups and by race/ethnicity.

## AUTHOR CONTRIBUTIONS

**Chun R Chao:** Data curation (equal); funding acquisition (equal); methodology (lead); supervision (equal); writing – original draft (lead). **Jessica Chubak:** Methodology (lead); writing – original draft (supporting). **Elisabeth F Beaber:** Methodology (supporting); project administration (lead); supervision (supporting); writing – review and editing (equal). **Aruna S Kamineni:** Funding acquisition (equal); methodology (supporting); writing – review and editing (lead). **Connie Mao:** Methodology (supporting); writing – review and editing (equal). **Michael Silverberg:** Data curation (equal); funding acquisition (equal); methodology (supporting); supervision (equal); writing – review and editing (equal). **Jasmin Anita Tiro:** Data curation (equal); funding acquisition (equal); methodology (supporting); supervision (equal); writing – review and editing (equal). **Celette S. Skinner:** Methodology (supporting); writing – review and editing (equal). **Michael P Garcia:** Data curation (supporting); formal analysis (lead); writing – review and editing (supporting). **Douglas A Corley:** Funding acquisition (equal); writing – review and editing (equal). **Rachel L Winer:** Writing – review and editing (equal). **Tina Raine‐Bennett:** Writing – review and editing (equal). **Sarah Feldman:** Writing – review and editing (equal). **Cosette M. Wheeler:** Data curation (equal); funding acquisition (equal); methodology (lead); supervision (equal); writing – review and editing (lead).

## FUNDING INFORMATION

This work was supported by the National Cancer Institute (NCI)‐funded Population‐based Research Optimizing Screening through Personalized Regimens (PROSPR I) consortium (grant numbers U54CA163262‐04S1, U54CA163261‐04S1, U54CA163308‐04S1, U54CA164336, and U01CA163304). The overall aim of PROSPR I is to conduct multi‐site, coordinated, transdisciplinary research to evaluate and improve cancer screening processes. The 10 PROSPR Research Centers reflect the diversity of US delivery system organizations.

## CONFLICT OF INTEREST

In 2021, Dr. Chun Chao was the Principal Investigator of a contract from Merck & Co., Inc. awarded to the Kaiser Permanente Southern California on ovarian cancer management.

From 2019 to 2020, Dr. Jessica Chubak was the Principal Investigator of a contract from Amgen, Inc. awarded to the Kaiser Foundation Health Plan of Washington to evaluate the accuracy of using electronic health record data to identify individuals with reduced ejection fraction heart failure. Dr. Cossette M. Wheeler has received funds from grants cooperative agreements related to cervical cancer screening and triage through her institution, the University of New Mexico. Cossette M. Wheeler reports receiving reagents and equipment from Roche Molecular Systems, Roche/Ventana Medical Systems, Hologic and Genera Biosystem through their institution and outside of the submitted work, research support from Becton Dickinson (BD) and Hologic through her institution and outside of the submitted work, personal fees from BD also outside of the submitted work, personal fees from BD also outside of this work. Dr. Sarah Feldman authored chapters for Up‐to‐date and received honorarium for this activity.

## Supporting information


Appendix S1

Table S1

## Data Availability

PROSPR data are available for collaboration and sharing after appropriate approvals and agreements are completed. Additional details are provided at: https://healthcaredelivery.cancer.gov/prospr/datashare.html
