# Supplementary material for: Gaps in the screening process for women diagnosed with cervical cancer in four diverse US health care settings
Source: Cancer Med. 2022 Sep 15;12(3):3705–17. doi: 10.1002/cam4.5226 (PMC9939213; doi:10.1002/cam4.5226)
Supplement: Supplementary file 1 — Appendix S1 [file CAM4-12-3705-s002.docx]

**Supplemental Material:**

**Detailed algorithm to assign care gap type in the screening process for cervical cancer**

**Cases [based on 2012 ASCCP management guidelines]**

1. **Treatment failed to prevent cancer**: was there any precancer treatment event in the intervention window?

If yes 🡪 Treatment failed to prevent

If no 🡪

1. **Gap in obtaining timely precancer treatment**: Was there a colposcopy procedure that took place at least 3 months prior to the end of the lookback period, and the colposcopy finding was CIN2+, and there was no treatment within 3 months after that colposcopy?

If yes 🡪 Gap in obtaining timely precancer treatment

If no 🡪

1. **Colposcopy failed to detect precancer**: Was there >=1 colposcopy and the colposcopy finding was <CIN2?

If yes 🡪 Colposcopy failed to detect

If no 🡪

1. **Gap in obtaining timely colposcopy after abnormal screening results** [defined as >3 months after test result]: Was there a screening test that took place at least 3 months prior to the end of the lookback period, and the test result required a colposcopy follow-up, but there is no colposcopy within 3 months following the test results [defined below] that require colposcopy follow up
   - 1. for those **age 21-24** **at time of Pap**:
        1. Pap>LSIL; or
        2. if there were three consecutive Paps (all three Paps have to take place at least 3 months prior to the end of the lookback period), the first and third one with results in [ASCUS or LSIL], and the second one with result in [normal, ASCUS, or LSIL] (colposcopy is needed after the third Pap)
        3. If there was [ASCUS & HPV+ or LSIL& HPV+] in co-test or HPV-reflex, followed by a pap of [normal, ASCUS or LSIL], followed by a pap of [≥ASCUS] (all three Paps have to take place at least 3 months prior to the end of the lookback period, colposcopy is needed after the third Pap)
        4. If none of above but there were two consecutive Paps with unsatisfactory specimen adequacy
     2. for those **age 25 and older** **at the time of Pap**:
        1. ASCUS & HPV+ or LSIL&HPV+ or any Pap > LSIL regardless of HPV result if co-test or HPV reflex test; Pap >= LSIL if Pap only; or
        2. if there were two consecutive co-test (both of co-tests have to take place at least 3 months prior to the end of the lookback period), first one with result in [Pap normal & HPV+, or Pap LSIL&HPV-, or ASCUS & HPV-], and the second one with result [Pap >=ASCUS or HPV+]. (colposcopy is needed after the second co-test)

or

1. if none of the above but there were two consecutive Pap with unsatisfactory specimen adequacy

If yes 🡪 Gap in obtaining timely colposcopy

If no 🡪

1. **Screening test failure – The results of the first test in the lookback period that did not meet the criteria for immediate colposcopy referral - co-testing or HPV reflex test results requiring repeated testing rather than immediate colposcopy referral**

**This should be anyone who did not fit (1) through (4) and**

- - 1. For those **age 21-24** at time of first Pap in the lookback period:
       1. ASCUS or LSIL or normal if Pap only, HPV reflex or co-test; or
       2. Pap with unsatisfactory specimen
    2. For those **age 25 and older** at the time of first Pap or first co-test in the lookback period:
       1. ASCUS or normal if Pap only;
       2. [Pap normal & HPV negative], [Pap normal &HPV+], [ASCUS & HPV negative], or [LSIL & HPV negative] if co-test or HPV-reflex test;
       3. If co-test, Pap-negative and HPV result unknown
       4. Pap with unsatisfactory specimen if Pap only or co-test

**Ignore the first screening test and look for the next screening test if:**

If Pap only: Pap result is missing (just missing, not due to unsatisfactory specimen)

If co-test: Pap result is missing, or HPV result is missing.

If there is no other screening test or if the next screening test is within 3 months before the end of the lookback period, then this woman will be excluded from the Table 3 tabulation. Please provide the numbers of such women.

1. **Lack of on-time screening:** Was there any screening test in the lookback period?

If no 🡪 Lack of on-time screening

If yes 🡪 Was there a screening test within the first 3 years and 3 months (to allow a 3-month buffer) in the lookback period?

If no 🡪 **Lack of on-time screening**

If yes 🡪

1. **No clear failure**: There was no clear failure in the screening continuum in the lookback period.
