# Supplementary material for: Gaps in the screening process for women diagnosed with cervical cancer in four diverse US health care settings
Source: Cancer Med. 2022 Sep 15;12(3):3705–17. doi: 10.1002/cam4.5226 (PMC9939213; doi:10.1002/cam4.5226)
Supplement: Supplementary file 2 — Table S1 [file CAM4-12-3705-s001.docx]

**Supplemental Table. Distribution of potential care gaps and care gap types in the screening history among women diagnosed with cervical cancer at PROSPR I cervical sites.**

| **Care Gap Type**  Specific care gap | **Site 1**  **n = 148** | **Site 2**  **n = 113** | **Site 3**  **n = 77** | **Site 4**  **n = 161** | **Total**  **N = 499*** |
| --- | --- | --- | --- | --- | --- |
| **Diagnostic/Treatment Failure** | **49 (33.1%)** | **30 (26.5%)** | **6 (7.8%)** | **26 (16.1%)** | **111 (22.2%)** |
| Treatment of precancer failed to prevent cancer | 15 (10.1%) | <5† | 0 (0%) | <5† | 20 (4.0%) |
| Gap in obtaining precancer treatment | <5† | <5† | <5† | <5† | 6 (1.2%) |
| Colposcopy failed to detect precancer | 17 (11.5%) | 15 (13.3%) | <5† | 12 (7.5%) | 46 (9.2%) |
| Gap in follow-up with colposcopy | 15 (10.1%) | 12 (10.6%) | <5† | 9 (5.6%) | 39 (7.8%) |
| **Screening Test Failure** | **53 (35.8%)** | **44 (38.9%)** | **19 (24.7%)** | **40 (24.8%)** | **156 (31.3%)** |
| **Lack a Screening Test** | **45 (30.4%)** | **39 (34.5%)** | **52 (67.5%)** | **95 (59.0%)** | **231 (46.3%)** |
| **No Clear Care Gap** | <5† | **0 (0%)** | **0 (0%)** | **0 (0%)** | <5† |

PROSPR= Population-based Research Optimizing Screening through Personalized Regimens.

*Six women had missing results for Pap test, thus were excluded from this table as it was not possible to correctly assign their care gap.

Note that the percentage for the care gap types represents the sum of the percentage of the care gaps within that type. Percentages of the care gap types (in bold) add to 100%.

†Actual number not shown when cell size <5 for site-specific data.
